# Long-term outcome of Bartter syndrome in 54 patients: A multicenter study in Korea

**DOI:** 10.3389/fmed.2023.1099840

**Published:** 2023-03-13

**Authors:** Naye Choi, Seong Heon Kim, Eun Hui Bae, Eun Mi Yang, Keum Hwa Lee, Sang-Ho Lee, Joo Hoon Lee, Yo Han Ahn, Hae Il Cheong, Hee Gyung Kang, Hye Sun Hyun, Ji Hyun Kim

**Affiliations:** ^1^Department of Pediatrics, Seoul National University College of Medicine, Seoul, Republic of Korea; ^2^Department of Pediatrics, Seoul National University Children's Hospital, Seoul, Republic of Korea; ^3^Department of Internal Medicine, Medical School, Chonnam National University, Gwangju, Republic of Korea; ^4^Department of Pediatrics, Chonnam National University Hospital, Gwangju, Republic of Korea; ^5^Department of Pediatrics, Yonsei University College of Medicine, Seoul, Republic of Korea; ^6^Department of Internal Medicine, Kyung Hee University Hospital at Gangdong, Seoul, Republic of Korea; ^7^Department of Pediatrics, Ulsan University Asan Medical Center, Seoul, Republic of Korea; ^8^Kidney Research Institute, Seoul National University Medical Research Center, Seoul, Republic of Korea; ^9^Department of Pediatrics, Hallym University Sacred Heart Hospital, Anyang, Republic of Korea; ^10^Wide River Institute of Immunology, Seoul National University, Hongcheon, Republic of Korea; ^11^Department of Pediatrics, College of Medicine, St. Vincent's Hospital, The Catholic University of Korea, Seoul, Republic of Korea; ^12^Department of Pediatrics, Seoul National University Bundang Hospital, Seongnam, Republic of Korea

**Keywords:** Bartter syndrome, long-term outcome, failure to thrive, chronic kidney disease, nephrocalcinosis, inherited hypokalemia

## Abstract

**Introduction:**

Bartter syndrome (BS) is a rare salt-wasting tubulopathy caused by mutations in genes encoding sodium, potassium, or chloride transporters of the thick ascending limb of the loop of Henle and/or the distal convoluted tubule of the kidney. BS is characterized by polyuria, failure to thrive, hypokalemia, metabolic alkalosis, hyperreninemia, and hyperaldosteronism. Potassium and/or sodium supplements, potassium-sparing diuretics, and nonsteroidal anti-inflammatory drugs can be used to treat BS. While its symptoms and initial management are relatively well known, long-term outcomes and treatments are scarce.

**Methods:**

We retrospectively reviewed 54 Korean patients who were clinically or genetically diagnosed with BS from seven centers in Korea.

**Results:**

All patients included in this study were clinically or genetically diagnosed with BS at a median age of 5 (range, 0–271) months, and their median follow-up was 8 (range, 0.5–27) years. Genetic diagnosis of BS was confirmed in 39 patients: 4 had *SLC12A1* gene mutations, 1 had *KCNJ1* gene mutations, 33 had *CLCNKB* gene mutations, and 1 had *BSND* mutation. Potassium chloride supplements and potassium-sparing diuretics were administered in 94% and 68% of patients, respectively. The mean dosage of potassium chloride supplements was 5.0 and 2.1 mEq/day/kg for patients younger and older than 18 years, respectively. Nephrocalcinosis was a common finding of BS, and it also improved with age in some patients. At the last follow-up of 8 years after the initial diagnosis, 41% had short stature (height less than 3rd percentile) and impaired kidney function was observed in six patients [chronic kidney disease (CKD) G3, *n* = 4; CKD G5, *n* = 2].

**Conclusion:**

BS patients require a large amount of potassium supplementation along with potassium-sparing agents throughout their lives, but tend to improve with age. Despite management, a significant portion of this population exhibited growth impairment, while 11% developed CKD G3–G5.

## 1. Introduction

Bartter syndrome (BS) is a rare salt-wasting tubulopathy with a prevalence of 1 in 100,000 (1). It is characterized by polyuria, hypokalemia, hypochloremic metabolic alkalosis, hyperreninemia, and hyperaldosteronism (2). BS has two classifications based on the clinical features: antenatal BS (aBS), a severe form presenting polyhydramnios, premature birth, severe salt and water loss, vomiting, diarrhea, failure to thrive, and nephrocalcinosis from birth (3, 4), and classic BS (cBS), typically presenting in infancy with hypokalemia, muscle weakness, and growth retardation (5, 6). BS is caused by mutations of genes that encode sodium, potassium, or chloride transporters in the thick ascending limb of the loop of Henle and/or the distal convoluted tubule. At present, BS is classified according to the causative gene (7): BS1, with *SLC12A1* encoding the sodium-potassium-chloride cotransporter in thick ascending limb of the loop of Henle; BS2, with *KCNJ1* of the luminal K+ channel ROMK; BS3, with *CLCNKB* of the basolateral chloride channel CIC-Kb; BS4a, with *BSND* encoding the beta-subunit for CIC-Ka and CIC-Kb; or BS4b, with simultaneous mutations in *CLCNKA* and *CLCNKB* (8–11). Some patients with BS3 present with Gitelman-like syndrome (GLS) characterized by hypocalciuria and mild hypomagnesemia, similar to the Gitelman syndrome (12). Recently, mutations of the *MAGED2* encoding melanoma-associated antigen D2 have been classified as BS5, characterized by severe polyhydramnios and preterm birth [<37 weeks of gestational age (GA)], but symptoms resolve after delivery without aggressive treatment (13). BS1, BS2, BS4, and BS5 are usually aBS, while BS3 is a cBS due to *CLCNKB*, but the phenotype does not always predict causative genes (14).

Though the presenting symptoms and initial management of BS are relatively well known, long-term outcomes and treatment are not sufficiently studied (14–17). To ameliorate the electrolyte imbalance of BS, a large amount of electrolyte supplementation is often necessary. Does this need for medication ever improve? Nephrocalcinosis is common for BS (2); does this impair kidney function in the long-term? Due to severe salt losing, patients with BS often present with failure to thrive; does it led to growth impairment when the patients age? Kidney function in BS is usually preserved, but patients with BS could develop chronic kidney disease (CKD) or kidney failure; the risk factors of progression to CKD have not been fully identified (14, 15, 18, 19). How many BS patients of our cohorts would progress to CKD? To answer these questions, in this study, we aimed to investigate the long-term clinical findings of patients with BS.

## 2. Materials and methods

Through a survey for the members of Genetic Kidney Disease Working Group of the Korean Society of Nephrology, Korean patients clinically or genetically diagnosed with BS were recruited from seven medical centers. The clinical diagnosis of BS was based on the judgment of each clinician, but most were made when a patient presented with hypokalemia, hypochloremic metabolic alkalosis, and renal salt wasting (20). The genetic diagnosis was mostly based on Sanger sequencing. We retrospectively reviewed their medical records between 1992 and 2020 for presenting symptoms, laboratory findings, genotype, medication, and their final height and kidney function. The estimated glomerular filtration rate (eGFR) was calculated using the bedside Schwartz equation (21) for children and CKD-EPI equation (22) for adults. BS was classified into aBS, cBS, and GLS according to phenotype, and five types (BS1–5) according to genotype. Genetic variants were classified as nontruncated when patients harbor missense mutation in any alleles, and truncated when patients have nonsense or frameshift variant in both alleles. Variants were identified for homozygocity or hemizygocity by a multiplex ligation-dependent probe amplification (MLPA) method for detecting large heterozygous deletion and/or DNA sequencing of the parents’ samples when the patient’s electropherogram showing a single peak in considerable patients. Some of the patients were previously described in the literature (11). Impaired kidney function (or CKD) was defined as an eGFR_cr_ < 60 mL/min/1.73 m^2^ in this study.

The presence of nephrocalcinosis was evaluated by kidney ultrasonography. The Institutional Review Board Seoul National University Hospital (IRB No. 2003-091-1109), and each participating center approved the protocols used in this study. The acquisition of permission was waived due to the retrospective nature of this study.

### 2.1. Statistical analysis

Descriptive variables were presented as median or mean [range or interquartile range (IQR)] or frequency counts (percent). Missing data were excluded for statistical analysis. The Mann–Whitney *U*-test was used to compare the genetic variant type (truncated group vs. nontruncated group) for rank values. Paired *t*-test or Wilcoxon signed rank test was performed to compare laboratory tests of initial and last visits. Chi-squared test or Fisher exact test was performed to compare binominal values such as the presence or absence of CKD, nephrocalcinosis, and growth impairment. We used SPSS software version 25.0 (Chicago, IL, United States) and GraphPad Prism Software (La Jolla, CA, United States). *p*-value of 0.05 was used as a threshold for statistical significance.

## 3. Results

### 3.1. Patient summary and clinical characteristics at diagnosis

**Figure 1:**
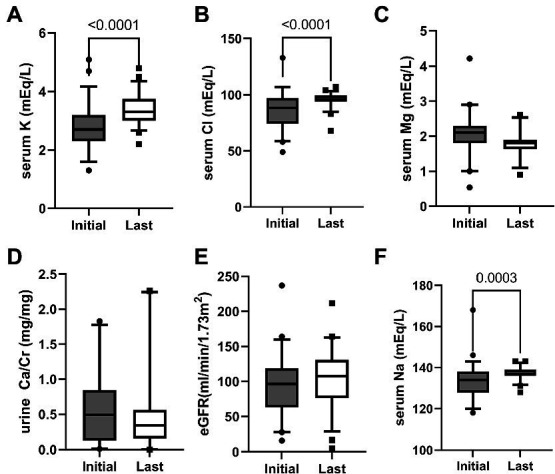
Chemistry after median 8 years follow-up. Box plots showing the quartiles, the 5th and 95th percentiles (whiskers) and extreme values. *p*-value is indicated on the graph for serum sodium, potassium, chloride, magnesium, estimated glomerular filtration rate (eGFR), and urine calcium/creatinine (Ca/Cr).

A total of 54 unrelated Korean patients (M:F = 33:21) were analyzed in this study (Table 1). None of the participants were offspring of consanguineous parents. Family history was identified in seven patients (13%). They were diagnosed with BS at a median age of five (range, 0–271) months old and followed up for 8 (median; range, 0.5–27) years. Failure to thrive was the most common presenting symptom, followed by polyuria, muscle cramping, and lethargy. Perinatal history of polyhydramnios, prematurity, and small for GA were not well documented, but found in 9%–47% when reported. Most of the patients showed hypokalemia and hypochloremic metabolic alkalosis on diagnosis (Figure 1). Notably, initial mean serum chloride level in BS3 patients was significantly lower than other patients (81.2 mEq/L vs. 94.5 mEq/L, *p* = 0.002). The hypomagnesemia (serum magnesium level < 1.5 mEq/L) was confirmed in six patients at the first visit: four were BS3 and two did not have mutations. Nephrocalcinosis was observed in 41% of the patients at the first visit. At presentation, height was compromised (height < 3rd percentile) in approximately half of the patients, and 72% were underweight (body weight < 3rd percentile). Notably, developmental delay was observed in eight patients (15%).

**Table 1:** Clinical characteristics according to genetic causes of Bartter syndrome patients.

	*SLC12A1* (*n* = 4)	*KCNJ1* (*n* = 1)	*CLCNKB* (*n* = 33)	*BSND* (*n* = 1)	Mutation negative or not available (*n* = 15)	Total (*n* = 54)
Male: Female (*n*)	2:2	1:0	18:15	1:0	11:4	33:21
Age of onset (median, month) (range)	11 (0–17)	12	4.5 (0–271)	0	18 (0–111)	5 (0–271)
Perinatal history						
Small for GA, % (*n*)	0	0	11 (3/28)	0	9 (1/11)	9 (4/45)
Preterm infant, % (*n*)	100 (4)	100 (1)	3 (1/29)	100 (1)	55 (6/11)	28 (13/46)
Polyhydramnios, % (*n*)	100 (4)	100 (1)	36 (8/22)	100 (1)	40 (4/10)	47 (18/38)
Initial symptom						
Failure to thrive, % (*n*)	50 (2)	100 (1)	76 (25)	100 (1)	27 (4)	6 (33)
Polyuria, % (*n*)	50 (2)	100 (1)	12 (4)	100 (1)	13 (2)	19 (10)
Lethargy, % (*n*)	0	100 (1)	12 (4)	0	13 (2)	13 (7)
Muscle cramping, % (*n*)	0	0	15 (5)	0	13 (2)	13 (7)
Clinical presentation, % (*n*)						
Antenatal BS	24 (1)	100 (1)	30 (10)	100 (1)	20 (3)	30 (16)
Classic BS	75 (3)	0	67 (22)	0	73 (11)	67 (36)
Gitelman-like syndrome	0	0	3 (1)	0	7 (1)	3 (2)
Family history, % (*n*)	25 (1)	100 (1)	9 (3)	0	13 (2)	13 (7)
**eGFR, mean (range), and clinical manifestations**						
On the first visit						
eGFR, mL/min/1.73m^2^	104.4 (69.9–125.0)	16.1	99.3 (27.7–237.0)	83.0	87.2 (34.6–156.0)	94.1 (16.1–237.0)
SNHL, % (*n*)	0	0	3 (1)	100 (1)	7 (1)	6 (3)
Nephrocalcinosis, % (*n*)	100 (4)	100 (1)	21 (6/29)	100(1)	83 (5/6)	41 (17/41)
On the last visit						
eGFR, ml/min/1.73m^2^	76.6 (74.5–80.8)	63.3	107.6 (5.0–164.0)	88.6	106.1 (17.0–211.8)	103.9 (5.0–211.8)
Impaired kidney function, % (n)	0 (0)	0	12 (4)	0 (0)	13 (2)	11 (6)
SNHL, % (*n*)	0	0	6 (2)	100 (1)	7 (1)	7 (4)
Nephrocalcinosis, % (*n*)	100 (4)	100 (1)	13 (3/24)	100(1)	46 (6/13)	35 (15/43)
Developmental delay, % (*n*)	25 (1)	0	12 (4)	100 (1)	13 (2)	15 (8)

BS, Bartter syndrome; GA, gestational age; eGFR, estimated glomerular filtration rate [calculated using the bedside Schwartz equation for children and CKD-EPI equation for adults]; Impaired kidney function, defined as an eGFR_cr_ < 60 mL/min/1.73 m^2^; SNHL, Sensorineural hearing loss.

### 3.2. Clinical characteristics according to genetic variants

Of 48 patients who underwent genetic testing, 39 were confirmed to have genetic causes of BS, primarily *CLCNKB* (83%, *n* = 33). None of the patients in this study were classified as BS5. The clinical characteristics according to genetic causes are described in Table 1. All patients with BS1, BS2, and BS4 were born as preterm and had prenatal history of polyhydramnios, but the birth weight was appropriate for GA (weight between 10th and 90th percentiles). However, 11% of the BS3 patients were small for GA (birth weight < 10th percentile for GA). Nephrocalcinosis was noted in all patients with genetically confirmed BS of non-*CLCNKB* genes and 21% of BS3. Sensorineural hearing loss (SNHL) was noted in three patients, of whom one had *BSND* mutation and one had *CLCKNB* mutation, diagnosed during infancy. All these three patients visited the hospital before 1 month of age because of polyuria or vomiting.

Of total 33 patients confirmed by *CLCNKB* variants, detailed genotypes for three patients were not available. 23 of 30 (76.7%) patients showed truncated variants (Supplementary Tables S1, S2). Patients with truncated vs. nontruncated variants did not significant differences in initial height, weight, or electrolyte at the first presentation or last follow-up (Supplementary Table S1). The most common genetic variant was W610 X (c.1830G > A in exon 16) in our cohort (40%–50%; 6 alleles were uncertain due to absence of MLPA test). Detailed genotypes by Sanger sequencing of BS (36 patients) are described in Supplementary Table S2.

### 3.3. Changes in biochemical parameters and clinical data during follow-up

**Figure 2:**
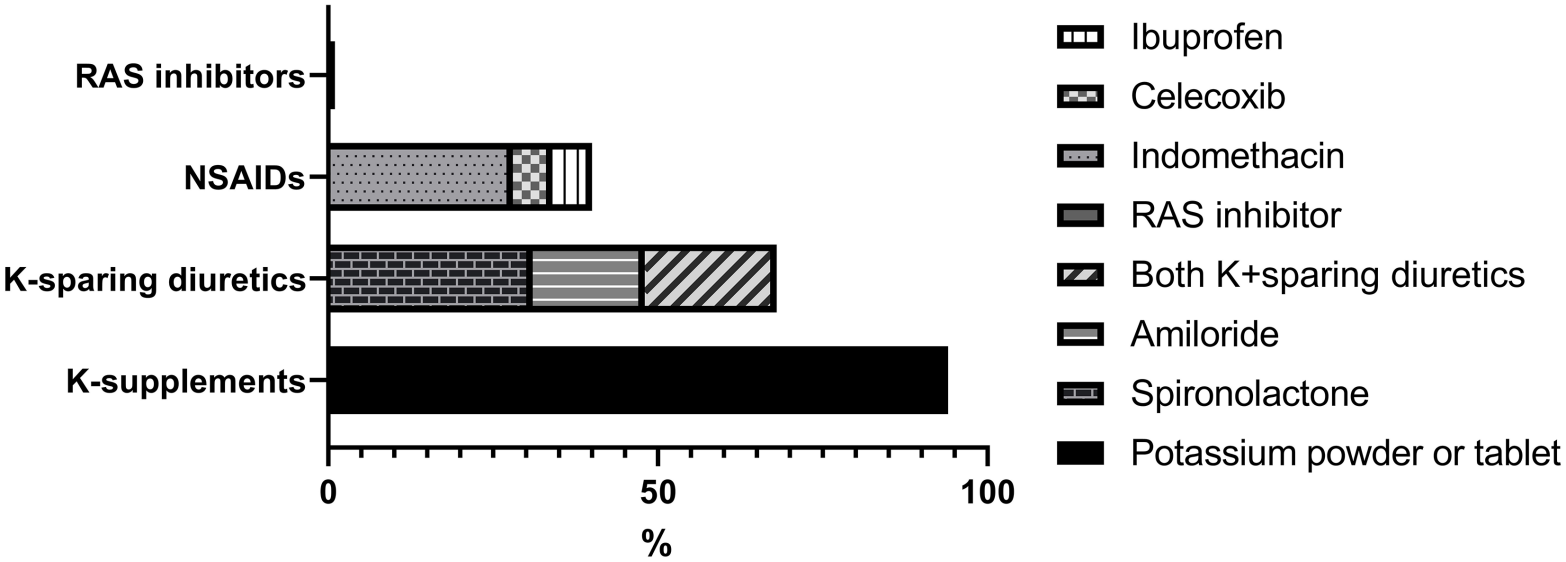
Medical treatment at the last follow-up. RAS, Renin-angiotensin system; NSAIDs, non-steroidal anti-inflammatory drugs; K, potassium.

After follow-up for a median of 8 years, serum sodium, potassium and chloride levels increased significantly compared to the initial findings (Figure 1) with prescription depicted in Figure 2. At their mean age of 14.90 (range, 1–31) years, 94% of the patients were taking potassium chloride supplements of 4.30 mEq/day/kg (body weight range, 0.01–14.13 mEq/day/kg). The mean dosage of potassium chloride supplements was 5.00 (range, 0.01–14.12) mEq/day/kg for those under the age of 18, and 2.06 (range, 0.51–5.2) mEq/day/kg (*p* = 0.056) for patient older than 18 years, and even two patients discontinued the medication. Potassium-sparing diuretics were administered in 68% of patients, while sodium chloride supplements were prescribed to 26%. A total of 33 patients (61%) had taken nonsteroidal anti-inflammatory drugs (NSAIDs), and 21 (39%) continued until the last follow-up. Renin-angiotensin-system (RAS) inhibitor had been prescribed in five patients (enalapril in three, captopril in one, and losartan in one). At the last follow-up, only one patient (5 years old) was treated with RAS inhibitor, and 80% of the patients had plasma K ≥ 3.0 mEq/L. Other laboratory findings including eGFR_cr_, blood renin, aldosterone and urinary calcium excretion did not significantly change after treatment.

#### 3.3.1. Outcomes of kidney function

**Figure 3:**
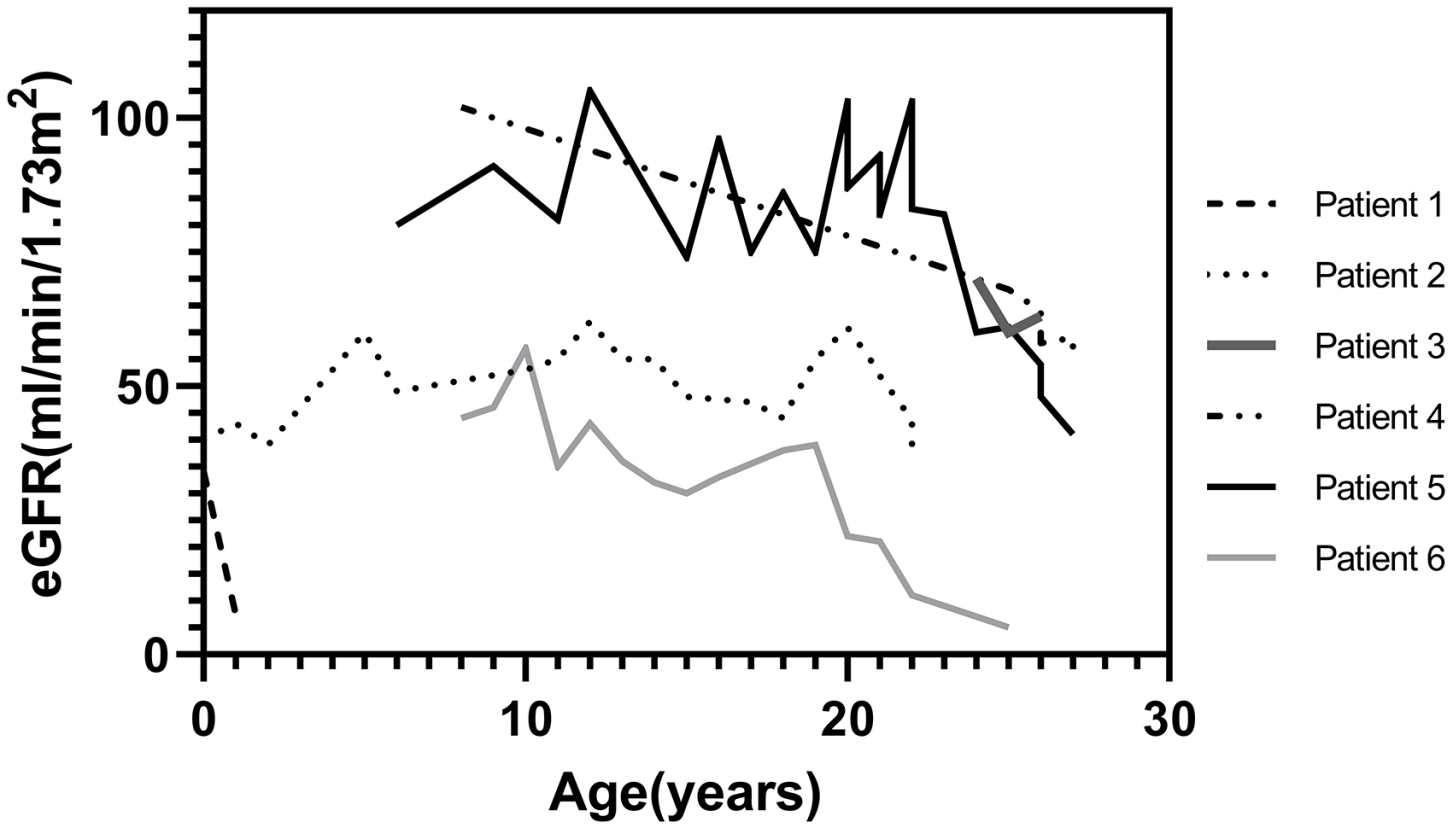
Change of eGFR of six patients with kidney impairment at the last follow-up (Bartter syndrome type 3: patient 2, 3, 5, 6). eGFR, estimated glomerular filtration rate [calculated using the bedside Schwartz equation for children and CKD-EPI equation for adults]; Kidney impairment, defined as an eGFR_cr_ < 60 mL/min/1.73 m^2^. No variants were found in patient 1 and 4.

However, impaired kidney function was observed in six patients (CKD G3 [eGFR, 30–59 ml/min/1.73 m^2^], *n* = 4); (CKD G5 [eGFR <15 mL/min/1.73 m^2^ or treated by kidney replacement therapy], *n* = 2); four were BS3 and two showed no variants (Figure 3). Half of them already showed impaired kidney function at the first visit. Kidney pathology was examined in one patient who later required kidney replacement therapy and was diagnosed with focal segmental glomerulosclerosis. Comparison of clinical characteristics at the first and last visits of all patients with BS were described in Supplementary Table S3. Regarding risk factors of CKD, nephrocalcinosis was noted in one patient and NSAIDs have been prescribed in three patients. Except for the current age (impaired kidney function group: median age was 28 [IQR, 22–29] years vs. (normal kidney function group: median age was 13 [IQR, 8–17] years, *p* < 0.001), clinical characteristics were not significantly different between those with and without impaired kidney function.

#### 3.3.2. Outcomes in nephrocalcinosis and SNHL

Nephrocalcinosis disappeared in four patients (BS3, *n* = 3; no mutation, *n* = 1) and newly noted in other four patients including two patients of BS3 (genotypes of the other two were not identified). Two patients with BS3 who had nephrocalcinosis at diagnosis had no follow-up data. Overall, nephrocalcinosis was observed in 35% (15/43) of evaluated patients at the last visit; 100% in BS1 (4/4), BS2 (1/1), and BS4 (1/1) and 12.5% in BS3 (3/24). Patients with and without nephrocalcinosis did not have significant differences.

In our study, SNHL was newly detected in one patient during follow-up period, resulting in a total four (7%) patients at the last follow-up (*BSND* mutation, *n* = 1; *CLCKNB* mutation *n* = 2).

#### 3.3.3. Outcomes of development and growth

In this cohort, we identified eight patients (15%) with developmental delay as at the first visit. Growth impairment (height < 3rd percentile) was noted in 22 of 54 patients (41%) at the last follow-up, including three patients who had been treated with growth hormone (GH). For patients older than 19 years (*n* = 10), their median heights were 163.6 (range, 160.9–170.0) cm for male and 158.7 (range, 150.0–175.8) cm for female, and 50% (5/10) had a short stature. However, our results did not demonstrate significant differences in age at presentation, genetic type, eGFR, nephrocalcinosis, initial urinary calcium excretion, serum sodium and chloride level between patients with short stature and those with normal growth. Additionally, NSAIDs usage or potassium supplement was not associated with growth. In our cohort, a total of nine patients were treated with GH and one-third of them achieved growth gain. Of three patients tested for GH deficiency, only one was positive.

## 4. Discussion

In this study, we analyzed the clinical and molecular characteristics of 54 Korean patients with BS through long-term follow-up (median of 8 years). At the last follow-up, most patients still required a large amount of potassium supplementation along with potassium-sparing agents to ameliorate their electrolyte imbalance, but the amount was decreased. We found that growth impairment was noted in 41% and CKD in 11% of the cohort. We summarized the comparison of genetic and clinical outcomes in different cohorts of patients with Bartter syndrome in Supplementary Table S4.

The genetic characteristics of BS vary depending on ethnic differences and each study (11, 18, 23). In this study, the most common affected gene was *CLCNKB* as previously reported from United Kingdom and Japan (18, 24). This is different from French or German study, gene as *SLC12A1* or *KCNJ1*, respectively (14, 23).

Similar to the literature, *CLCNKB* mutations might present aBS or GLS as well (14, 25, 26). Different from previous study (14), the higher proportion of BS3 patients with cBS phenotype in our study may be due to lack of genetic diagnosis, where some BS3 patients with GLS might be simply managed as Gitelman syndrome without genetic study (27). Also, the onset age of aBS was not always antenatal as previously reported (28–30). Among the *CLCNKB* mutations, the most common genetic variant was W610 X (c.1830G > A in exon 16) in our cohort, which accounted for approximately half, consistent with Japan (24). This variant might be a founder mutation in both similar ethnicities. Our study, compared to previous studies, did not find significant differences in phenotypes according to variant type (truncating vs. nontruncating) (14, 31, 32), Insufficient number of patients and the ethinic differences may affect such a result.

In this study, initial mean serum chloride level in BS3 patients was significantly lower than in other patients, as previously reported, due to the distribution of the CLC-Kb channel throughout the thick ascending limb, distal convolute tubule, and early collecting duct (14, 33, 34).

The main goal of BS treatment is to correct hypokalemia and prevent dehydration by using potassium supplements, sodium supplements, NSAIDs, and sometimes additional potassium-sparing diuretics (16). However, there is little data available about the duration and dosage of medications in BS patients, and there are concerns about using potassium-sparing diuretics (20). The dose of potassium is not exactly known, but a target level of plasma potassium may be 3.0 mEq/L (20). Kaur et al. (18) observed that potassium supplementation decreased after 1 year of age (peak for 1 year; mean, 6.6 mEq/day/kg) and that at the age of 15 years, the dose of potassium decreased to 1.5 mEq/day/kg, consistent with our study. Therefore, the need for electrolyte supplementation may decrease as the patient gets older, even not in all cases. Considering the significant role of overactivated prostaglandins in the pathogenesis of BS, the use of NSAIDs might help (35). There were no differences in clinical outcomes for each drug (ibuprofen, celecoxib, or indomethacin) in this study, as previously recommended (20). Routine usage of potassium-sparing agent (spironolactone, eplerenone, or amiloride), angiotensin-converting enzyme inhibitor, or angiotensin receptor blocker to ameliorate hypokalemia is not recommended because these medications can exacerbate renal salt wasting and polyuria (20, 35).

Complications of BS include nephrocalcinosis, SNHL, and rarely developmental delay (18, 20). Nephrocalcinosis has been reported to be associated with BS1 and BS2, but this study revealed that nephrocalcinosis can also be found in patients with BS3, consistent with Japan study, but higher (14, 24). In our study, nephrocalcinosis was found in 13%–41% of patients, similar to Japan study (24). Notably, the final eGFR was not significantly different in patients with and without nephrocalcinosis, inconsistent with the United Kingdom study (18). For treatment, to improve nephrocalcinosis, indomethacin could be administered in the neonatal period (30), but not thiazide because it can cause life-threatening hypovolemia in BS patients and potassium citrate can exacerbate metabolic alkalosis. Therefore, there is no specific way to treat nephrocalcinosis in BS patients except for hydration.

SNHL identified in 4 patients was, as expected, noted in the patient with *BSND* mutation (7); however, the other two genetically confirmed patients with SNHL had *CLCNKB*, and as *CLCNKA* was studied only in one patient, so the patient not tested for *CLCNKA* might be a BS4b case. SNHL in BS3 has been reported in two cases of BS3 (36, 37), therefore suggesting that hearing impairment is another symptom of BS3. Regarding developmental delay, our cohort has eight cases. Intellectual performance of BS patients is usually normal, but some developmental delayed cases have been reported in previous study (18, 38).

Growth impairment is a common complication is BS patients (18), and this can be due to a number of causes such as electrolyte imbalance, polyuria, poor feeding, and CKD. Treatment with indomethacin (17) and growth hormone therapy may help to improve growth. GH deficiency has also been reported in patients with BS (39–42), most frequently in patients with BS3 (15). Growth retardation does not improve even after correction of serum potassium level, but growth response to GH therapy was excellent. On the other hand, administration of GH had no effect until COX inhibitor administration (15). Therefore, it is suggested that correcting electrolyte imbalance and GH administration should be considered together. Still, the cause of growth failure is unclear (35), therefore further research is needed to determine the best treatment options.

Patients in this cohort had lower eGFR initially than at the last follow-up. We assumed that the reason was that they were mostly infants (median age of 5 months) and dehydrated on their first visit. Prevalence of impaired kidney function was 11% of patients in this cohort, while CKD (G3 or above) has been observed in 0%–64% of BS according to the literature (11, 14, 15, 18, 19, 43). Such a large discrepancy between the studies might come from the different proportion of each genotype, difference of follow-up period, or ethnic differences. The causes of kidney failure have been proposed as nephrocalcinosis, long-term treatment with NSAIDs, chronic hypokalemia and prematurity (15, 18, 44, 45). Regarding genotypes, progression to CKD was more severe in patients with BS1 and BS4 than BS2 and BS3 in previous reports (14, 20). In particular, the risk of CKD and its potential relationship to prolonged use of NSAIDs, chronic hypokalemia, and chronic hypovolemia (46) is not well documented in this study due to missing data. Moreover, in the case of causative gene, it was difficult to show a statistically significant differences between each genetic group due to small number.

The limitations of this study include small numbers, missing data, not fully covered genetic tests, lack of information for drug discontinuation, and retrospective nature. Also, this was a single ethnic cohort, which may have biased our results, although no significant differences were observed in affected genes when compared to other Western cohorts.

## 5. Conclusion

To the best of our knowledge, this is the only long-term follow-up study of Korean patients with BS. and found that that *CLCNKB* is the most common variant type. Similar to other studies of long-term outcome of BS, this study shows that *CLCNKB* is the most common variant type and potassium requirements improved with age. Nephrocalcinosis is a common finding of BS, and it also improved in some patients as they grew up. However, a correlation with impaired kidney function has not been confirmed. Due to severe salt losing, patients with BS often present with a failure to thrive, eventually leading to growth impairment in 41% of patients. However, the causes of growth impairment were not fully understood. According to literature, 7%–64% of BS patients progress to CKD, and we reported in 11% of this cohort. The risk factors of the progression to CKD remain unknown. Therefore, a large international prospective cohort study is required.

## Data availability statement

The raw data supporting the conclusions of this article will be made available by the authors, without undue reservation.

## Ethics statement

The studies involving human participants were reviewed and approved by Seoul National University Hospital. Written informed consent from the participants’ legal guardian/next of kin was not required to participate in this study in accordance with the national legislation and the institutional requirements.

## Author contributions

HGK and NC were primarily responsible for study conception and design. NC, HGK, HSH and JHK were primarily responsible for analysis and interpretation of data. NC, HGK, HSH and JHK drafted the article. NC, SHK, EHB, EMY, KHL, SHL, JHL, YHA, HIC, HGK, HSH, and JHK contributed to revisions and provided intellectual content of critical importance to the work. All authors contributed to the article and approved the submitted version.

## Funding

This work was supported by the National Research Foundation of Korea (NRF) grant funded by the Korea government (MSIT – Ministry of Science and ICT) (no. 2020R1A2C1100974).

## Conflict of interest

The authors declare that the research was conducted in the absence of any commercial or financial relationships that could be construed as a potential conflict of interest.

## Publisher’s note

All claims expressed in this article are solely those of the authors and do not necessarily represent those of their affiliated organizations, or those of the publisher, the editors and the reviewers. Any product that may be evaluated in this article, or claim that may be made by its manufacturer, is not guaranteed or endorsed by the publisher.
