# Corrigendum: Long-term outcome of Bartter syndrome in 54 patients: a multicenter study in Korea

**DOI:** 10.3389/fmed.2023.1225353

**Published:** 2023-05-31

**Authors:** Naye Choi, Seong Heon Kim, Eun Hui Bae, Eun Mi Yang, Keum Hwa Lee, Sang-Ho Lee, Joo Hoon Lee, Yo Han Ahn, Hae Il Cheong, Hee Gyung Kang, Hye Sun Hyun, Ji Hyun Kim

**Affiliations:** ^1^Department of Pediatrics, Seoul National University College of Medicine, Seoul, Republic of Korea; ^2^Department of Pediatrics, Seoul National University Children's Hospital, Seoul, Republic of Korea; ^3^Department of Internal Medicine, Medical School, Chonnam National University, Gwangju, Republic of Korea; ^4^Department of Pediatrics, Chonnam National University Hospital, Gwangju, Republic of Korea; ^5^Department of Pediatrics, Yonsei University College of Medicine, Seoul, Republic of Korea; ^6^Department of Internal Medicine, Kyung Hee University Hospital at Gangdong, Seoul, Republic of Korea; ^7^Department of Pediatrics, Ulsan University Asan Medical Center, Seoul, Republic of Korea; ^8^Kidney Research Institute, Seoul National University Medical Research Center, Seoul, Republic of Korea; ^9^Department of Pediatrics, Hallym University Sacred Heart Hospital, Anyang, Republic of Korea; ^10^Wide River Institute of Immunology, Seoul National University, Hongcheon, Republic of Korea; ^11^Department of Pediatrics, College of Medicine, St. Vincent's Hospital, The Catholic University of Korea, Seoul, Republic of Korea; ^12^Department of Pediatrics, Seoul National University Bundang Hospital, Seongnam, Republic of Korea

**Keywords:** Bartter syndrome, long-term outcome, failure to thrive, chronic kidney disease, nephrocalcinosis, inherited hypokalemia

In the published article, there was an error in affiliation 5. Instead of “Department of Pediatrics, Yonsei University Severance Children's Hospital, Seoul, Republic of Korea”, it should be “Department of Pediatrics, Yonsei University College of Medicine, Seoul, Republic of Korea”.

In the published article, an author name was incorrectly written as “Geum Hwa Lee”. The correct spelling is “Keum Hwa Lee”.

The authors apologize for this error and state that this does not change the scientific conclusions of the article in any way. The original article has been updated.

