# Supplementary material for: Long-term outcome of Bartter syndrome in 54 patients: A multicenter study in Korea
Source: Front Med (Lausanne). 2023 Mar 13;10:1099840. doi: 10.3389/fmed.2023.1099840 (PMC10040751; doi:10.3389/fmed.2023.1099840)
Supplement: Supplementary file 1 [file Table_1.docx]

**Supplemental Table S1. Differences of clinical characteristics according to mutation type in Bartter syndrome type 3 in this cohort**

|  | **Truncated group**  **(n=23)** | **Non-truncated group**  **(n=7)** | ***P* value** |
| --- | --- | --- | --- |
| Onset of symptoms (months) | 17.7 (0.0–271.0) | 3.0 (0.0–8.0) | 0.122 |
| **Initial index** |  |  |  |
| Height (cm, %) (< 3p) | 34.8 (8) | 71.4 (5) | 0.198 |
| Body weight (kg, %) (< 3p) | 78.3 (18) | 71.4 (5) | 0.612 |
| Serum sodium (mEq/L) | 132.6 (118.0–140.0) | 131.5 (126.0–141.0) | 0.774 |
| Serum potassium (mEq/L) | 2.7 (1.6–3.6) | 3.0 (2.5–3.4) | 0.158 |
| Serum chloride (mEq/L) | 83.1 (49.0–99.0) | 85.0 (65.0–96.0) | 0.631 |
| eGFR (mL/min/1.73m^2^) | 98.0 (48.6–151.2) | 84.5 (27.7–130.0) | 0.614 |
| **Last index** |  |  |  |
| Serum sodium (mEq/L) | 137.8 (133.0–142.0) | 137.2 (133.0–142.0) | 0.701 |
| Serum potassium (mEq/L) | 3.4 (2.6–4.8) | 3.5 (3.0–4.1) | 0.848 |
| Serum chloride (mEq/L) | 96.6 (89.0–101.0) | 97.2 (94.0–100.0) | 0.631 |
| eGFR (mL/min/1.73m^2^) | 101.0 (5.0–155.3) | 96.4 (42.8–126.0) | 0.611 |

We defined truncated group and non-truncated group when patients harbor truncating mutation in both alleles and when patients harbor missense mutation in any alleles, respectively.

Values are expressed as % (numbers) and mean (range). The *P* values are for comparisons of the two groups with the Mann-Whitney U test and Fisher’s exact test

p, percentile; eGFR, estimated glomerular filtration rate [calculated using the bedside Schwartz equation for children and CKD-EPI equation for adults]; Kidney impairment, defined as an eGFR_cr_ <60 mL/min/1.73 m^2^

**Supplemental Table S2. Sanger sequence data of Bartter syndrome (36 patients) in this cohort**

| **Patient ID** | **Variant 1** | **Variant 2** | **ACMG classification** |
| --- | --- | --- | --- |
| ***CLCNKB*** | |  |  |
| **Non-truncating mutations** | |  |  |
| P3 | c.647C>T; p.Pro216Leu | c.1309G>A; p.Gly437Arg | LP/LP |
| P6 | c.359G>T; p.Gly120Val | c.1332_1335del; p.Ser4453LeufsTer33 | Pathogenic/LP |
| P7 | c.1830G>A; p.Trp610Ter | c.647C>T; p.Pro216Leu | Pathogenic/LP |
| P10 | c.1830G>A; p.Trp610Ter | c.359G>T; p.Gly120Val | Pathogenic/Pathogenic |
| P21 | c.1830G>A; p.Trp610Ter | c.647C>T; p.Pro216Leu | Pathogenic/LP |
| P38 | c.647C>T; p.Pro216Leu | Total deletion | LP/ |
| P39 | Total deletion | c.359G>T; p.Gly120Val | /Pathogenic |
| **Truncating mutations** | |  |  |
| P1* | c.1830G>A; p.Trp610Ter | Homozygous or Large deletion | Pathogenic |
| P4 | c.1830G>A; p.Trp610Ter | Homozygous | Pathogenic |
| P12 | c.1830G>A; p.Trp610Ter | Homozygous | Pathogenic |
| P14 | c.1830G>A; p.Trp610Ter | c.633dup; p.Val212SerfsTer70 | Pathogenic/LP |
| P15 | c.1830G>A; p.Trp610Ter | c.633dup; p.Val212SerfsTer70 | Pathogenic/LP |
| P16 | Exon 1-14 deletion | Total deletion |  |
| P17 | Total deletion | c.863del; p.Leu288ArgfsTer61 | /LP |
| P23 | c.1830G>A; p.Trp610Ter | Total deletion | Pathogenic |
| P24^§^ | c.118delA; p.Arg40GlyfsTer4 | c.659G>A:p.Trp220Ter | Pathogenic/Pathogenic |
| P26 | c.1830G>A; p.Trp610Ter | c.1830G>A; p.Trp610Ter | Pathogenic |
| P29 | c.1830G>A; p.Trp610Ter | Total deletion | Pathogenic |
| P31* | c.1830G>A; p.Trp610Ter | Homozygous or Large deletion | Pathogenic |
| P33 | c.685dup; :p.Ser229PhefsTer53 | Exon 1-3 deletion | LP/ |
| P36 | Total deletion | Homozygous |  |
| P41 | Total deletion | Homozygous |  |
| P42* | c.1830G>A; p.Trp610Ter | Homozygous or Large deletion | Pathogenic |
| P43 | c.1830G>A; p.Trp610Ter | Total deletion | Pathogenic |
| P44* | c.1830G>A; p.Trp610Ter | Homozygous or Large deletion | Pathogenic |
| P45* | c.1830G>A; p.Trp610Ter | Homozygous or Large deletion | Pathogenic |
| P46* | c.1830G>A; p.Trp610Ter | Homozygous or Large deletion | Pathogenic |
| P47 | c.334C>T; p.Gln112Ter | Homozygous | LP |
| P48 | c.1830G>A; p.Trp610Ter | Homozygous | Pathogenic |
| P53 | c.1830G>A; p.Trp610Ter | Homozygous | Pathogenic |
| ***SLC12A1*** |  |  |  |
| P18 | c.1307G>A; p.Cys436Tyr | c.1679T>C; p.Leu560Pro | LP/LP |
| P28 | c.382C>T; p.Arg128Ter | c.1679T>C; Leu560Pro | Pathogenic/LP |
| P49 | Exon 1 deletion | Homozygous |  |
| P50 | c.1307G>A; p.Cys436Tyr | c.1561G>T; p.Ala521Ser | LP/LP |
| ***KCNJ1*** |  |  |  |
| P27 | c.140G>A; p.Gly47Glu | c.931C>T; p.Arg311Trp | LP/LP |
| ***BSND*** |  |  |  |
| P25 | c.139G>C; p.Gly47Arg | Homozygous | LP |

Abbreviation: ACMG, The American College of Medical Genetics and Genomics; LP, likely pathogenic; Hemi, hemizygous; homo, homozygous.

Notes:

- The reference sequences for each variant are as follows:

*CLCNKB:* NM_000085.5, hg38 except for the P24^§^ (NM_001165945.2)

*SLC12A1*: NM_000338.3, hg38; *KCNJ1*: NM_000220.6, hg38; *BSND*: NM_057176.3, hg38

- Pathogenicity of Mutations was referred to the varsome, intervar, clinvar databases.

- We defined truncated group and non-truncated group when patients harbor truncating mutation in both alleles and when patients harbor missense mutation in any alleles, respectively.

- *Variants were identified for homozygosity or hemizygosity by a multiplex ligation-dependent probe amplification method except for some patients (marked as a star) for detecting large heterozygous deletion and/or DNA sequencing of the parents’ samples when the patient’s electropherogram showing a single peak in the *CLCNKB* gene.

- Of total 33 patients confirmed by *CLCNKB* variants, detailed genotypes for three patients not listed in the table were not available.
