# Supplementary material for: Long-term outcome of Bartter syndrome in 54 patients: A multicenter study in Korea
Source: Front Med (Lausanne). 2023 Mar 13;10:1099840. doi: 10.3389/fmed.2023.1099840 (PMC10040751; doi:10.3389/fmed.2023.1099840)
Supplement: Supplementary file 4 [file Table_4.DOCX]

**Supplementary Table S4.** **Comparison of genetic and clinical outcomes in different cohorts of patients with Bartter syndrome**

| **Study** | **Nation** | **Total** | **Type of mutations  (in BS3 only)** | **FU period (median)** | **Nephrocalcinosis** | **Growth impairment*** | **Developmental delay** | **Sensorineural hearing loss** | **Chronic kidney disease**** |
| --- | --- | --- | --- | --- | --- | --- | --- | --- | --- |
| Amrit K. et. al. (20) | UK | 34 (68%: non-Caucasian)  (BS3: 9 of 18 pts) | NA | 15 yrs | OFVt: 41% (BS3: 0%) | (5 yrs) 42%,  (10 yrs) 35%,  (15 yrs) 43% | NA | OFVt: 12% (BS3: 0%) | (15yrs) 64% |
| Seys E. et al. (15) | France | * Study only for BS3 *  115, follow-up for 77 pts | Truncating: 45% | 8 yrs | At Dx: 16%  OLVt: 18% (including stone) | OLVt: 18% | OLVt: 10% | NA | OLVt: 12% (G3, 3%; G4, 1%; G5, 8%) |
| Garcia Castano A (21) | Spain | * Study only for BS3 *  30 (87%: Spanish), FU for 15 pts | Truncating: 13%  (m/c) A204T (80%) | 19 yrs | At Dx: 20%  OLVt: 20% (including stone) | OLVt: 0% | NA | NA | OLVt: 0% |
| Han, Y. et al. (34) | China | 42 (BS3: 36) | Truncating: 83% | 21 months | NA | OFVt: 48%  OLVt: 12% | NA | NA | OFVt and OLVt: 0% |
| Nozu, K.et al. (26) | Japan | 13 (BS3: 9) | Truncating: 100%  (m/c) W610X (56%) | Baseline data only | OFVt: 38% (BS3: 11%) | NA | NA | NA |  |
| Matsunoshita, N. et al. (29) | Japan | 30 (all BS3) | NA | Baseline data only | NA | NA | NA | NA | At Dx (mean 4 yrs): 17% (G3) |
| This study | Korea | 54 (BS3: 33) | Truncating: 77%  (m/c) W610X (40-50%) | 8 yrs | OFVt: 41% (BS3: 21%)  OLVt: 35% (BS3: 13%) | OFVt: 48%  OLVt: 41% | OLVt: 15% (BS3: 12%) | OFVt: 6% (BS3: 3%)  OLVt: 7% (BS3: 4%) | OLVt: 11% (G3, 7%; G5, 4%) |

*It was defined by height less than 3 percentile (or below -2SD).

** It is defined by estimated glomerular filtration rate < 60 ml/min/1.73m^2^ [calculated using the bedside Schwartz equation for children and CKD-EPI equation for adults]

(Stage of CKD are defined as follows: G3, eGFR 30-59; G4, eGFR 15-29; G5, eGFR <15. eGFR, estimated glomerular filtration rate in mL/min/1.73m^2^)

We defined truncated group and non-truncated group when patients harbor truncating mutation in both alleles and when patients harbor missense mutation in any alleles, respectively.

Abbreviation: BS3, Bartter syndrome type 3; FU, follow-up; yrs, years; pts, patients; NA, not available; OFVt, on the first visit; OLVt, on the last visit; Dx, diagnosis
